# Hsa-miR-21-5p is induced by interleukin-6 and affects multiple pathogenic factors associated with fibroblast-like synoviocytes in rheumatoid arthritis

**DOI:** 10.1038/s41598-025-02840-z

**Published:** 2025-06-03

**Authors:** Kei Araki, Sho Mokuda, Hiroki Kohno, Naoya Oka, Hirofumi Watanabe, Michinori Ishitoku, Tomohiro Sugimoto, Yusuke Yoshida, Junya Masumoto, Shintaro Hirata

**Affiliations:** 1 Department of Clinical Immunology and Rheumatology, Hiroshima University Hospital, Hiroshima, 734- 8551 Japan; 2 Division of Laboratory Medicine, Hiroshima University Hospital, 1-2-3 Kasumi, Minami-ku, Hiroshima, 734-8551 Japan; 3 Department of Pathology, Ehime University Proteo-Science Center and Graduate School of Medicine, Shitsukawa, Toon, Ehime 791-0295 Japan

**Keywords:** Rheumatoid arthritis, Fibroblast-like synoviocytes, microRNA, hsa-miR-21-5p, Next-generation sequencing, Rheumatoid arthritis, Molecular medicine

## Abstract

Fibroblast-like synoviocytes (FLS) contribute significantly to the pathogenesis of rheumatoid arthritis (RA), particularly through their roles in synovitis and joint destruction. The microRNAs (miRNAs) are short non-coding RNA that regulate gene expression post-transcriptionally. Although more than 2000 human miRNAs are registered, comprehensive analyses of miRNA expression in FLS are limited. Herein, we investigated the relationship between miRNAs and FLS. Next-generation sequencing (small RNA-seq) was performed on primary cultured FLS derived from patients with RA to analyze the mature miRNA expression pattern. To assess inflammation-induced changes in miRNA levels, FLS were cultured with cytokines and evaluated by RT-qPCR. MiRNA mimics were transfected into FLS and an immortalized synovial fibroblast cell line (MH7A cells), and validated using conventional RNA-seq. Out of 2861 mature miRNAs, 297 mature miRNAs were detected and intronic miRNAs were predominated. Notably, hsa-miR-21-5p was abundantly expressed and its expression was enhanced by IL-6 stimulation. The induction of miR-21-5p mimic decreased the expression of Programmed Cell Death 4 (PDCD4) and Osteoprotegerin (OPG), while upregulating Semaphorin 5A (SEMA5A). MiR-21-5p mimic led to enhanced cell proliferation. These data suggest that hsa-miR-21-5p in FLS may exacerbate the pathophysiology of rheumatoid synovitis by promoting FLS proliferation, highlighting its potential as a therapeutic target in RA.

**Supplementary Information:**

The online version contains supplementary material available at 10.1038/s41598-025-02840-z.

## Introduction

Rheumatoid arthritis (RA) is characterized by synovial proliferation and synovitis that can lead to joint destruction. It is a heterogeneous disease caused by an abnormal autoimmune response, triggered by a combination of genetic and environmental factors that initiate and sustain synovial inflammation^1,2^. Fibroblast-like synoviocytes (FLS) in the synovial lesions of patients with RA play a crucial role in its pathogenesis^3,4^. FLS are characterized by intense proliferation and can contribute to the local production of cytokines (e.g., interleukin-6 [IL-6]), chemokines (e.g., C-C motif chemokine ligand 2 [CCL2], and C-X-C motif chemokine ligand 12 [CXCL12]), a classical inducer of osteoclast formation (e.g., receptor activator of NF-kappaB ligand [RANKL]), and proteolytic enzymes (e.g., matrix metalloproteinases) that degrade the extracellular matrix, as demonstrated by single-cell RNA-sequencing^3,5^. These features are associated with the development of RA pathophysiology such as synovitis and joint destruction^3–6^.

MicroRNAs (miRNAs) are 20–24 nucleotide single-stranded RNAs that are transcribed from genomic DNA to primary miRNAs (pri-miRNAs), processed to precursor miRNAs (pre-miRNAs), and cleaved into short RNA duplexes. One strand of mature miRNA functions by being incorporated into RNA-induced silencing complexes (RISCs) containing Argonaute proteins. These complexes bind to 3′-untranslated regions (UTR) of complementary sequences of mRNA transcripts and inhibit mRNA translation or directly cleave mRNA, thereby suppressing the expression of the target gene^7–11^. Some miRNAs are also reportedly expressed in FLS and function as regulators of RA pathophysiology. The expression of miR-146a/b was low in patients with osteoarthritis (OA) and normal tissues, whereas miR-146a/b was highly expressed in synovial fibroblasts of patients with RA. In addition, miR-146a/b levels were markedly upregulated in RA synovial fibroblasts when stimulated with tumor necrosis factor-alpha (TNF-α) or interleukin-1-beta (IL-1β)^12^. The expression of miR-155 is significantly higher in the synovial tissues of patients with RA than that in controls. MiR-155 suppresses the expression of forkhead box protein O3a (FOXO3a) as a target gene, resulting in the secretion of inflammatory cytokines such as IL-1β, IL-6, and TNF-α, and the proliferation of FLS^13^. Thus, multiple miRNAs have been reported to be involved in RA-FLS functions, such as proliferation and inflammation. Although more than 2,000 human microRNAs are registered in the database, to the best of our knowledge, few reports have comprehensively analyzed the expression patterns of miRNAs in RA-FLS.

In this study, we aimed to analyze the expression patterns and functions of miRNAs in RA-FLS. Among several candidate miRNAs, we investigated the function of cytokine-inducible miR-21-5p in RA-FLS. Our data showed that miR-21-5p promotes cell proliferation and multipotently regulates several arthritis-related factors.

## Results

### Distribution of MicroRNA in FLS

**Fig. 1:**
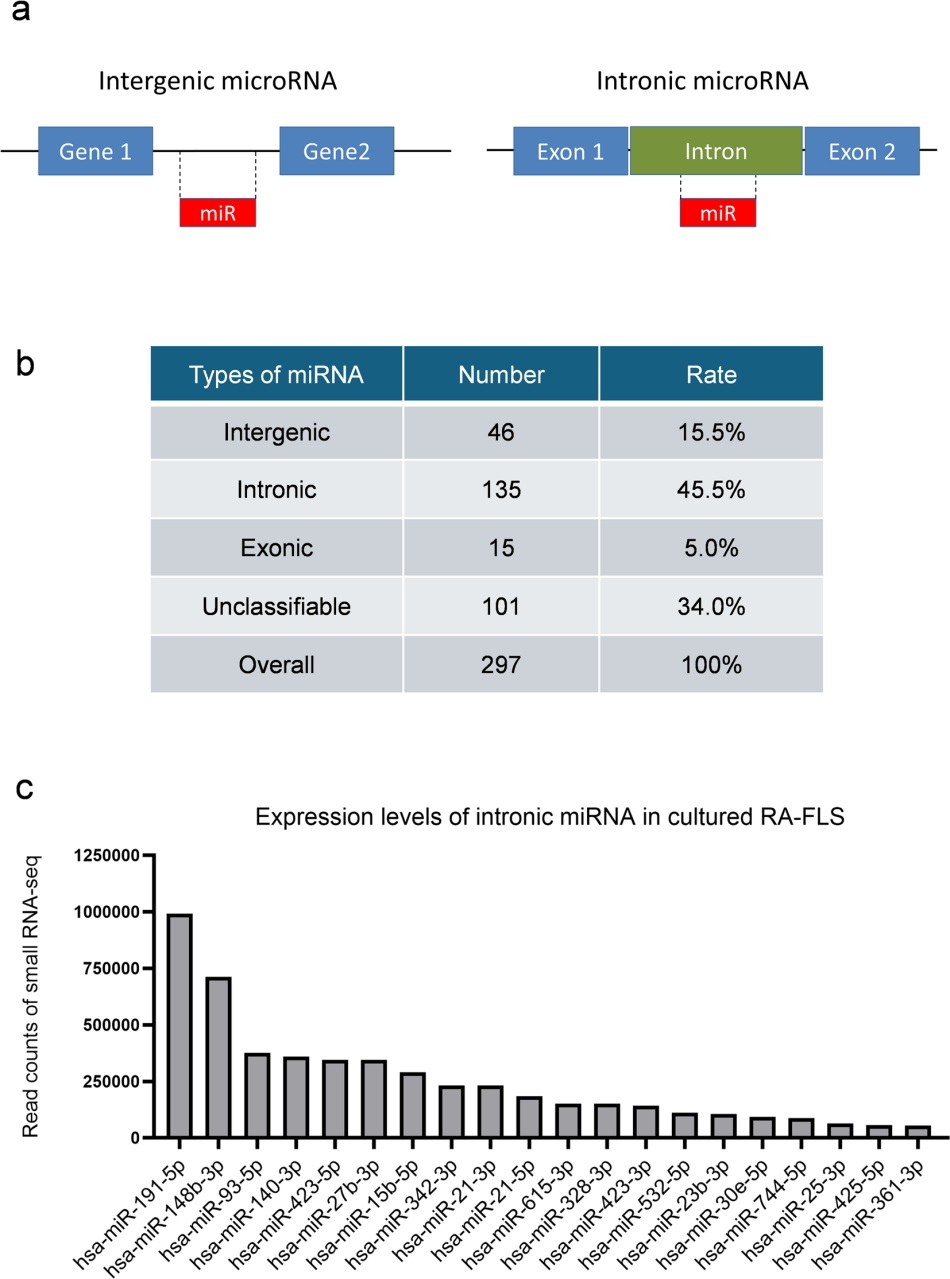
The intronic type is a large population of microRNAs expressed in FLS. (**a**) Schematics of intergenic and intronic miRNAs. (**b**,**c**) Small RNA-seq performed for 2861 mature miRNAs in fibroblast-like synoviocytes (FLS) obtained from patients with rheumatoid arthritis (RA) (*n* = 2, from each individual subject). 297 species of mature miRNAs were detected. (**b**) The distribution ratio for the types of miRNAs. 297 miRNAs were categorized by their respective types (e.g., intergenic, intronic, exonic, and an unclassifiable group). (**c**) Top 20 most highly expressed intronic miRNAs, displayed with read count of small RNA-seq.

The miRNAs can be classified into several groups according to their location: intergenic miRNAs, and intragenic miRNAs which mainly consist of intronic and exonic miRNAs^14^. Intergenic miRNAs are located independently between genes and frequently possess their own promoters (Fig. 1a). Intronic and exonic miRNAs are located within the intron and exon regions of the host genes, respectively. A large proportion of all human miRNAs is intragenic, and intronic miRNAs account for more than 85% of all intragenic miRNAs^15^. The characteristics of miRNA expression and classification in some cancer cells, such as colorectal and breast cancer cells, have been analyzed in detail^16^. The miRNAs are also expressed in FLS; however, the classification of miRNAs in FLS has never been reported. To clarify the distribution of miRNAs, we performed a small RNA-seq analysis which covered 15 to 40 nucleotides of non-coding RNAs, including mature microRNAs of about 22 nucleotides, and quantitative, not relative, lead counts could be obtained. Thus, small RNA-seq was performed for 2861 mature miRNAs in RA-FLS, which revealed that 297 mature miRNA species were detectable. The group of intronic miRNAs had the highest proportion (Fig. 1b and c). Supplementary Figure S1 shows the host genes for the top 20 most abundantly expressed mature intronic human miRNAs in RA-FLS.

### Inflammatory cytokine-induced alteration of microRNA expression levels in FLS

**Fig. 2:**
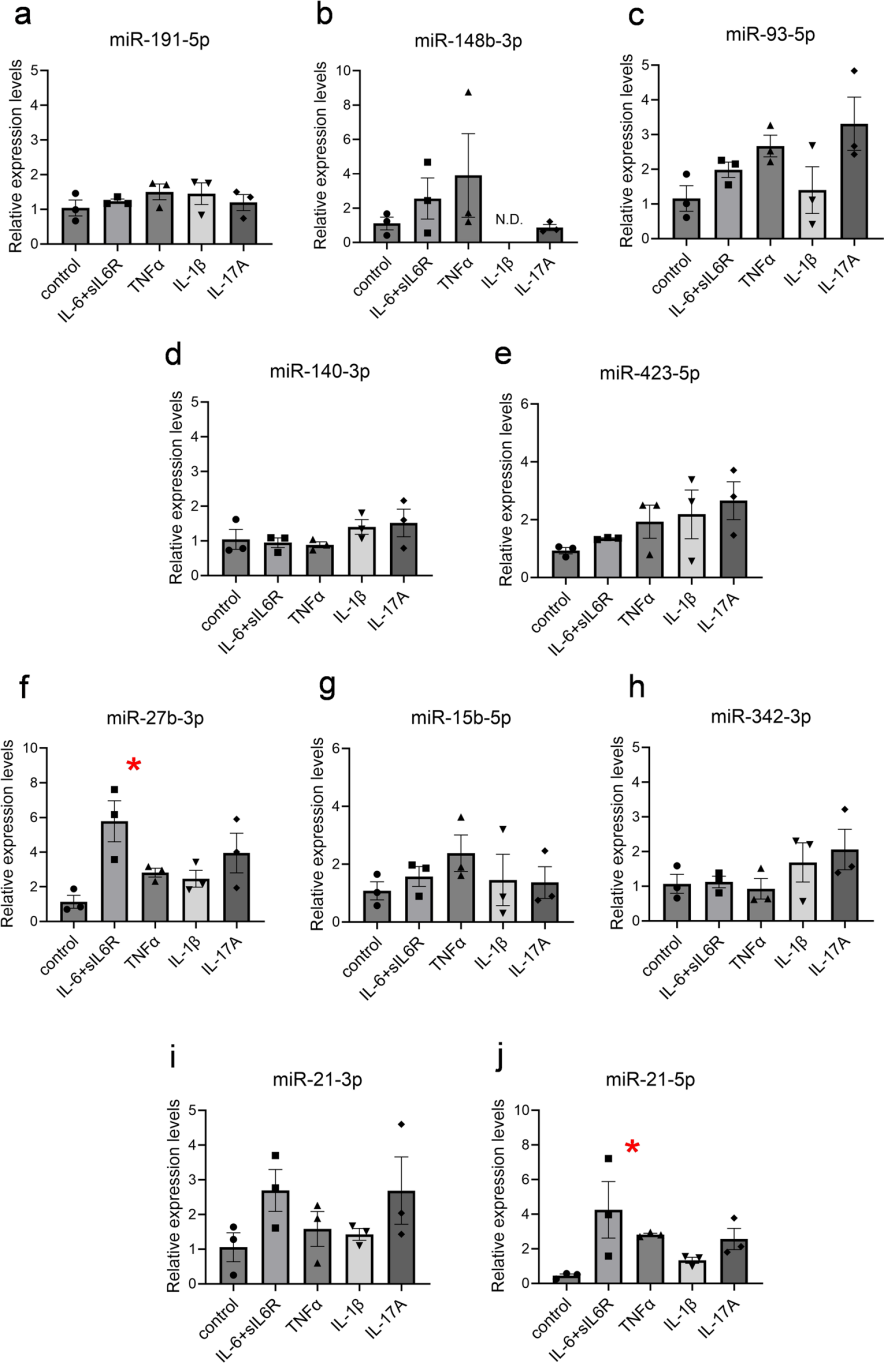
Alteration of intronic microRNA expression levels in cytokine-stimulated FLS. The FLS data were collected from patients with rheumatoid arthritis (RA). FLS were stimulated with IL-6 (100 ng/mL, ■) with 100 ng/mL of sIL-6R, TNFα (100 ng/mL, ▲), IL-1β (10 ng/mL, ▼), and IL-17 A (100 ng/mL, ◆) for 24 h. (**a**) hsa-miR-191-5p expression. (**B**) hsa-miR-148b-3p expression. (**c**) hsa-miR-93-5p expression. (**d**) hsa-miR-140-3p expression. (**e**) hsa-miR-423-5p expression. (**f**) hsa-miR-27b-3p expression. (**g**) hsa-miR-15b-5p expression. (**h**) hsa-miR-342-3p expression. (**i**) hsa-miR-21-3p expression (**j**) hsa-miR-21-5p expression. All expression levels were measured using RT-qPCR, and statistical analyses were performed using Dunn’s test (*p < 0.05). Data represent the mean ± the standard error of the mean (*n* = 3, from each individual subject).

Notably, intracellular miRNA concentrations can change rapidly^17^. In pathogenic synovial tissues, FLS are often affected by inflammatory conditions. We investigated whether the inflammatory cytokines affected the expression levels of intronic miRNAs. After RA-FLS were cultured under non-inflammatory conditions, these cells were stimulated with several recombinant proteins, such as IL-6/soluble IL-6 receptor alpha (sIL-6R), TNFα, IL-1β, and IL-17 A. Figure 2a–j shows that hsa-miR-27b-3p and hsa-miR-21-5p among the top 10 expressed intronic miRNAs were significantly upregulated by IL-6 and sIL-6R. In silico analysis using the TargetScan website (https://www.targetscan.org/vert_80/, accessed on October/20/2024) identified several theoretical target genes of hsa-miR-27a/b-3p. Subsequently, we examined the gene expression changes in miR-27b-3p mimic-transfected cells, such as an immortalized synovial fibroblast cell line (MH7A cells) and primary cultured FLS; however, we could not detect any significant changes in these genes (Supplementary Fig. S2).

The transcription of intronic miRNAs is often detected in parallel with the host gene expression^16,18^. However, the most highly expressed miRNAs in FLS did not move in parallel with the host gene expressions (Supplementary Fig. S3), indicating that these intronic miRNAs changes under cytokine stimulation were independent of host gene expression.

### Hsa-miR-21-5p expression is upregulated by IL-6 through STAT3 in the synovial tissues of patients with RA

**Fig. 3:**
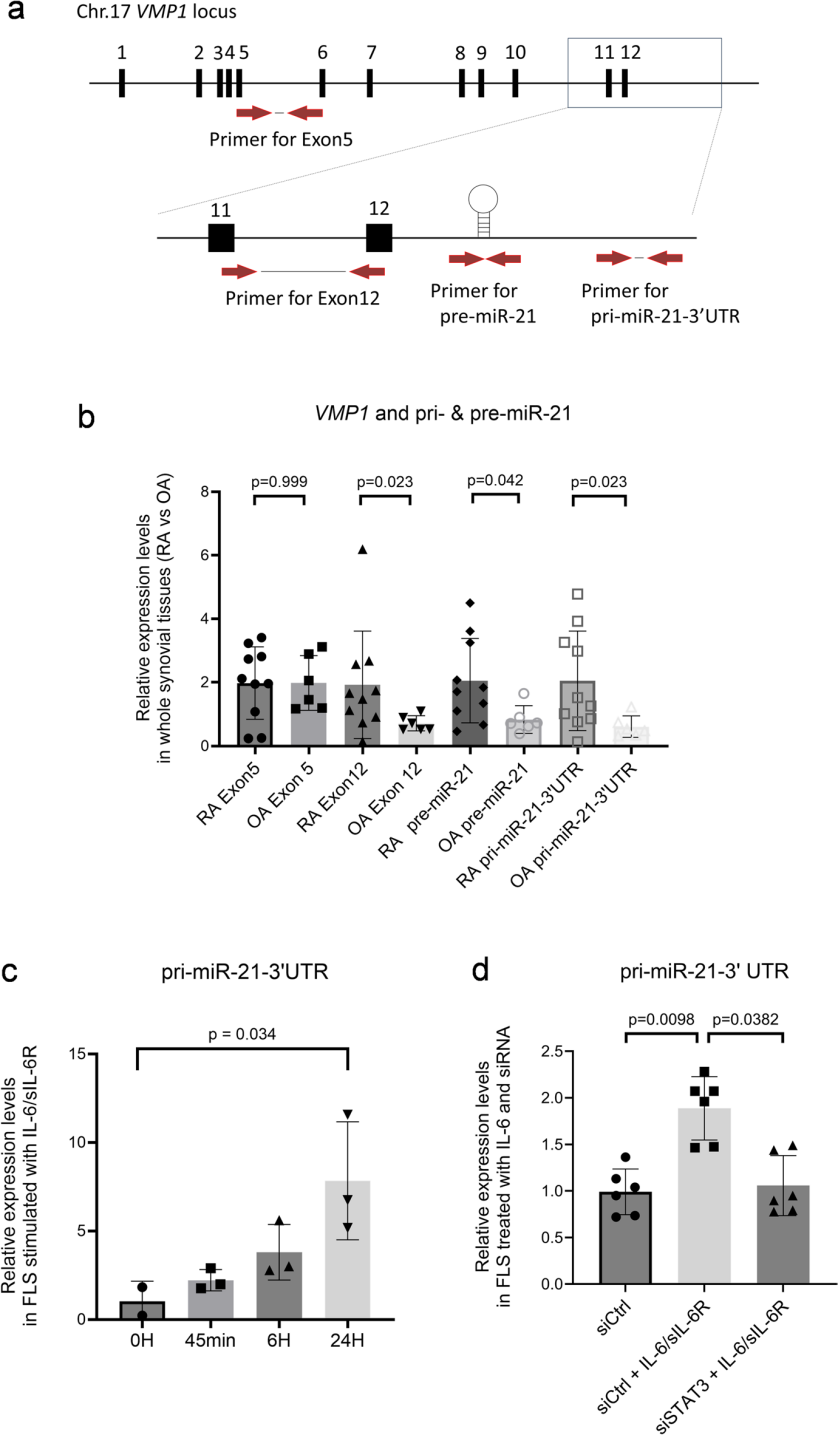
Pri-miR-21 in FLS is induced by IL-6 through STAT3, independent of host VMP1 expression. (**a**) The schema of the VMP1 gene locus on chromosome 17 in *Homo sapiens*. Red arrow indicates the location of PCR primers for Exon 5, Exon 12, pre-miR-21, and pri-miR-21-3’UTR. (**b**–**d**) Expression levels were measured using RT-qPCR. (**b**) Whole synovial tissues collected from patients with rheumatoid arthritis (RA; *n* = 10) and osteoarthritis (OA; *n* = 6). Data represent the mean ± the standard deviation, and statistical analysis was performed using U-test. (**c**, **d**) Pri-miR-21-3’UTR expression levels in fibroblast-like synoviocytes (FLS) collected from patients with RA. Data represents the mean ± the standard error of the mean, and statistical analysis was performed using Dunn test. (c) Time-dependent stimulation with recombinant IL-6 (100 ng/mL) and recombinant sIL-6R (100 ng/mL) (*n* = 3). (d) Stimulation with IL-6 (100 ng/mL) and sIL-6Rα (100 ng/mL) for 24 h, and small interfering RNA against STAT3 for 34 h (*n* = 6). VMP1, vacuole membrane protein 1.

The regulatory mechanism of miR-21-5p transcription has been reported to be stimulated by IL-6 and signal transduction and activator of transcription 3 (STAT3) in both human multiple myeloma cell lines and bovine cumulus cells^19,20^; however, it has never been reported in primary cultured FLS. The intron 10 of *vacuole membrane protein 1* (*VMP1)* has been described for miRNA proximal promoter region (miPPR)-21, and the 3’ UTR of Exon 12 encodes a 72-nucleotide hairpin containing mature miR-21 (pre-miR-21) and a non-functional region (pri-miR-21). Therefore, the expression of miR-21-5p can be induced independently of VMP1. To confirm these phenomena in the synovial tissues, we compared the specimens of RA and OA synovial tissues using reverse transcription-quantitative polymerase chain reaction (RT-qPCR) with specific primer sets (Fig. 3a). The expression levels of Exon 5 of *VMP1* were similar in RA and OA, whereas the expression levels of Exon12 of *VMP1*, pre-miR-21, and pri-miR-21 were higher in RA specimens compared with OA specimens (Fig. 3b). FLS, a major cell type in synovial tissue, was stimulated by IL-6, and subsequently exhibited time-dependent upregulation of pri-miR-21 (Fig. 3c). Moreover, this induction by IL-6 was suppressed by siRNA targeting STAT3 (Fig. 3d). Therefore, the synovial tissues and FLS express pri-miR-21 through IL-6 stimulation via STAT3 signaling.

### Hsa-miR-21-5p promotes FLS proliferation

**Fig. 4:**
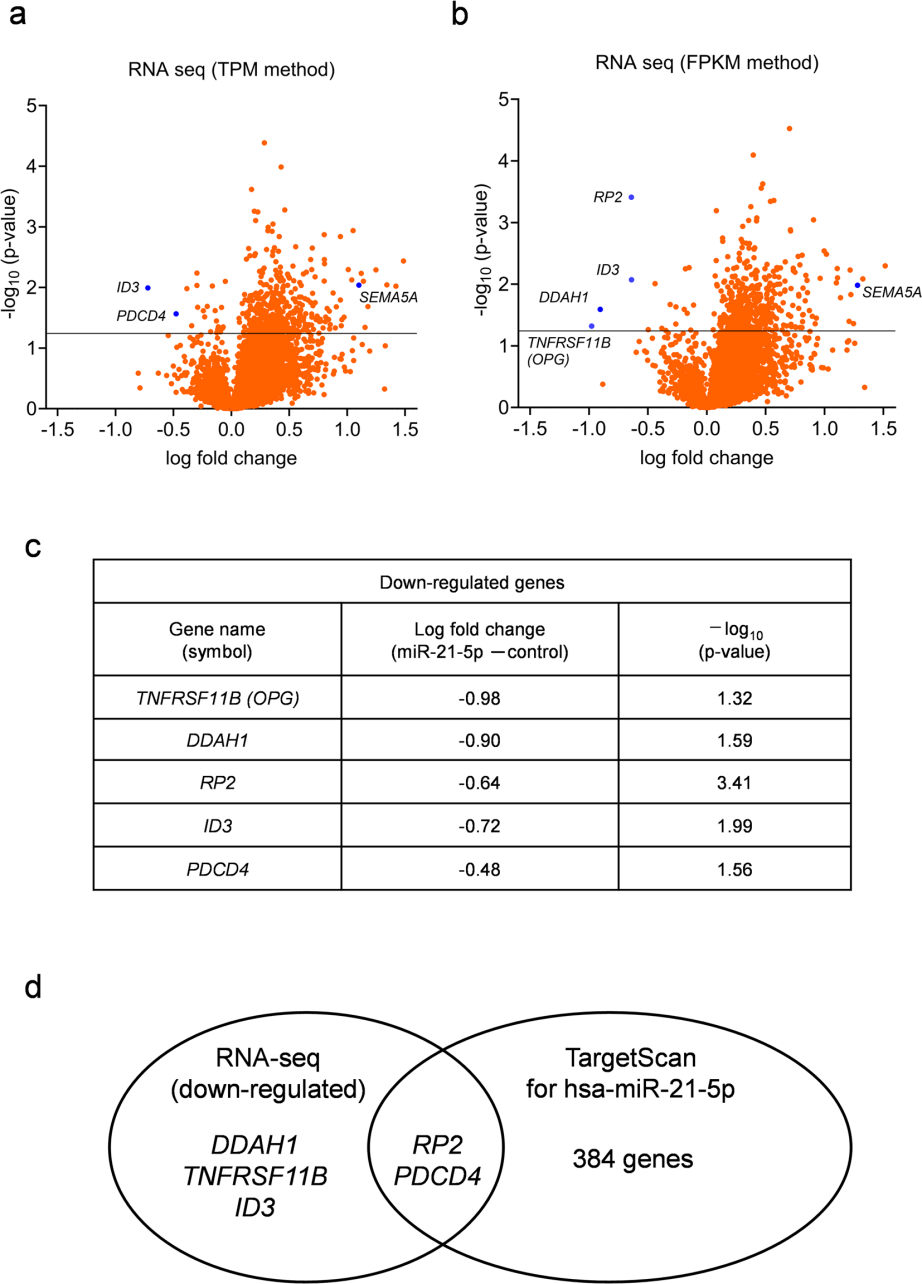
RNA-seq analysis of hsa-miR-21-5p-induced gene expression changes in FLS. RNA from miR-21-5p or miR control (miRNA negative control) mimic-transfected FLS was collected and analyzed using next-generation sequencing analysis (conventional RNA-seq) (*n* = 2, from each individual subject). (**a**) Volcano plot, analyzed with TPM method. (**b**) Volcano plot, analyzed with FPKM method. (**c**) Represents the top five downregulated genes. (**d**) The Venn diagram illustrates the relationship between downregulated genes detected with conventional RNA-seq analysis and genes that have potential target sequences for hsa-miR-21-5p, as predicted by the TargetScan database. DDAH1, dimethylarginine dimethylaminohydrolase 1; TNFRSF11B, TNF receptor superfamily member 11b; OPG, osteoprotegerin; RP2, retinitis pigmentosa 2; ID3, inhibitor of DNA binding 3; PDCD4, programmed cell death 4.

In patients with RA, plasma miR-21-5p was reportedly positively correlated with the disease activity score and ultrasound-detected synovial hypertrophy^21^, therefore, this miRNA may be one of the significant biomarkers for synovial proliferation. To elucidate the function of hsa-miR-21-5p in RA-FLS, conventional RNA-seq was performed on miR-21-5p mimic-transfected cells (Fig. 4a and b) (Supplementary Fig. S4, S5, and S6). Some of the most downregulated genes included *dimethylarginine dimethylaminohydrolase 1* (*DDAH1*), *TNF receptor superfamily member 11b* (*TNFRSF11B;* osteoprotegerin *[OPG]*), *retinitis pigmentosa 2* (*RP2*), *inhibitor of DNA binding 3* (*ID3*), and *programmed cell death 4* (*PDCD4*) (Fig. 4c). In silico targets for hsa-miR-21-5p searched in the TargetScan website (https://www.targetscan.org/vert_80/, accessed on November/10/2023) showed that the 3’UTR of *PDCD4* and *RP2* genes contain target sequences against miR-21-5p (Fig. 4d).

**Fig. 5:**
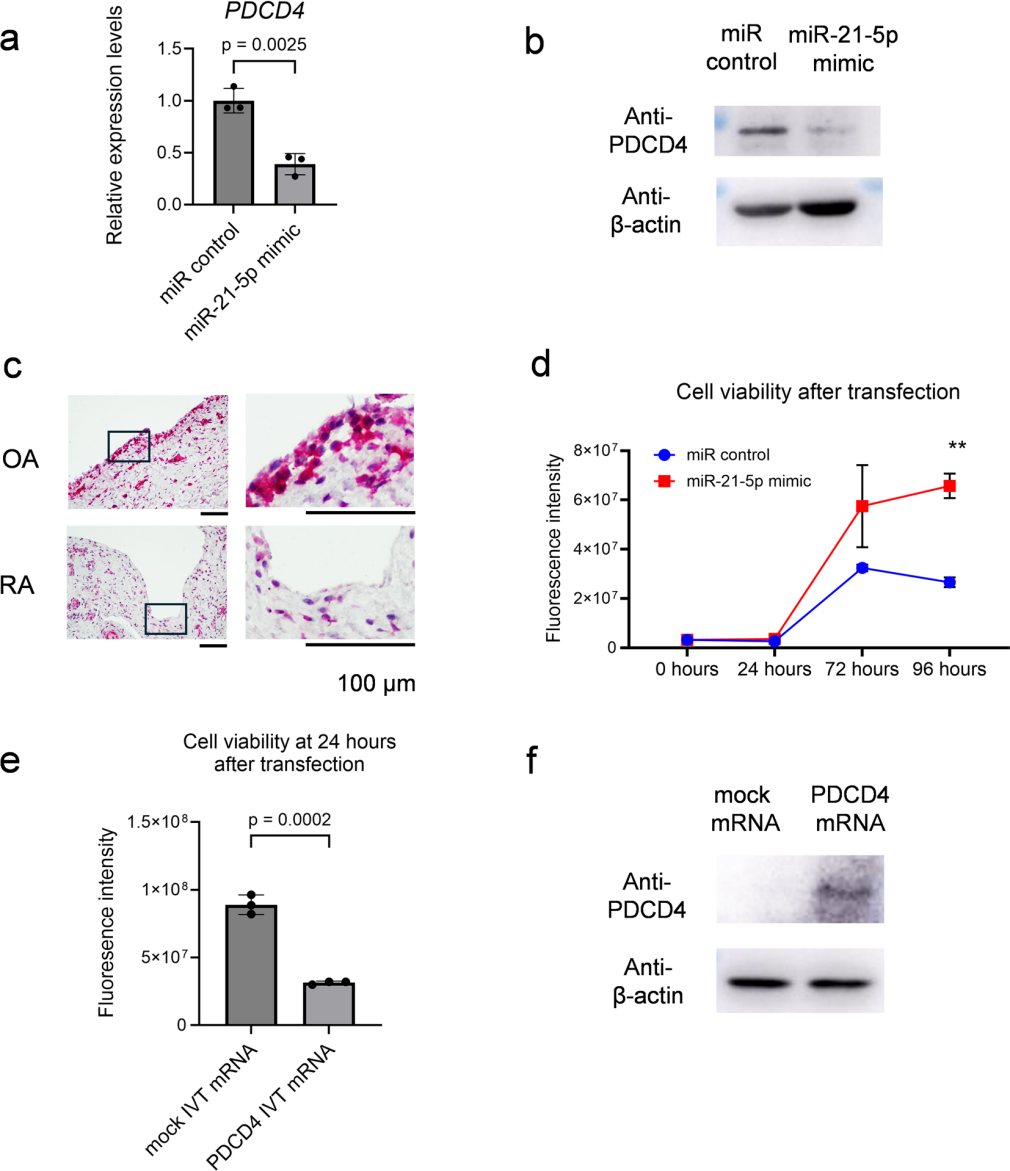
Hsa-miR-21-5p promotes fibroblast-like synoviocytes proliferation. (a, b) MH7A cells were transfected with miR-21-5p mimic or miR control. (**a**) *PDCD4* expression levels were measured using RT-qPCR (*n* = 3). (**b**) PDCD4 proteins were detected by Western blotting. (**c**) Immunohistochemistry (IHC) staining for PDCD4 in formalin-fixed paraffin-embedded (FFPE) synovial tissues which were harvested from patients with RA and OA (red, PDCD4; purple, hematoxylin). Black scale bar = 100 μm. (**d**) Cell viability assay using calcein-AM for MH7A cells transfected with miR-21-5p mimic or miR control (*n* = 3). (e, f) MH7A cells were transfected with PDCD4 IVT mRNA or mock IVT mRNA. (**e**) Cell viability assay using calcein-AM for MH7A cells at 24 h after transfection (*n* = 3). (**f**) PDCD4 proteins were assayed with Western blotting. (a, d, e) Data are presented as mean ± the standard error of the mean, t test. PDCD4, programmed cell death 4.

Subsequently, we conducted the following wet-lab experiments on these genes. In primary cultured FLS and MH7A cells, the miR-21-5p mimic suppressed PDCD4 expression (Figs. 4a and c and 5a and b). Conversely, a miR-21-5p inhibitor (Synthetic Tough Decoy [S-TuD] against miR-21-5p]) upregulated *PDCD4* expression (Supplementary Fig. S7). Furthermore, we found that PDCD4 was highly expressed in the synovial tissues of OA specimens compared with RA specimens, as detected by immunohistochemistry (IHC) (Fig. 5c). PDCD4 has been reported to be a target of hsa-miR-21-5p, mainly in the field of cancer research, such as breast and colorectal cancers^22,23^. Based on previous oncology reports, the suppression of PDCD4 by miR-21-5p may also be important in the pathophysiology of RA in FLS. The calcein assay showed that the miR-21-5p mimic promoted the viability of the FLS cell line (MH7A) compared with the miRNA negative control (miR control), in parallel with a decrease in PDCD4 expression (Fig. 5b and d). The relationship between PDCD4 and synovial fibroblast proliferation has not yet been reported. Then, additional data revealed that the induction of PDCD4 in vitro transcribed mRNA (IVT mRNA) in MH7A cells increased the PDCD4 protein levels and suppressed the cell viability (Fig. 5e and f). Therefore, hsa-miR-21-5p can directly promote FLS proliferation via PDCD4 suppression.

The miR-21-5p mimic also suppressed *RP2* expression (Fig. 4b and c). The RP2 expression in OA synovial tissues was higher than that in RA tissues, as detected by IHC (Supplementary Fig. S8). However, subsequent experiments did not demonstrate a relationship between RP2 and the pathophysiology of arthritis (Supplementary Fig. S9).

### Hsa-miR-21-5p could function multipotently in FLS

**Fig. 6:**
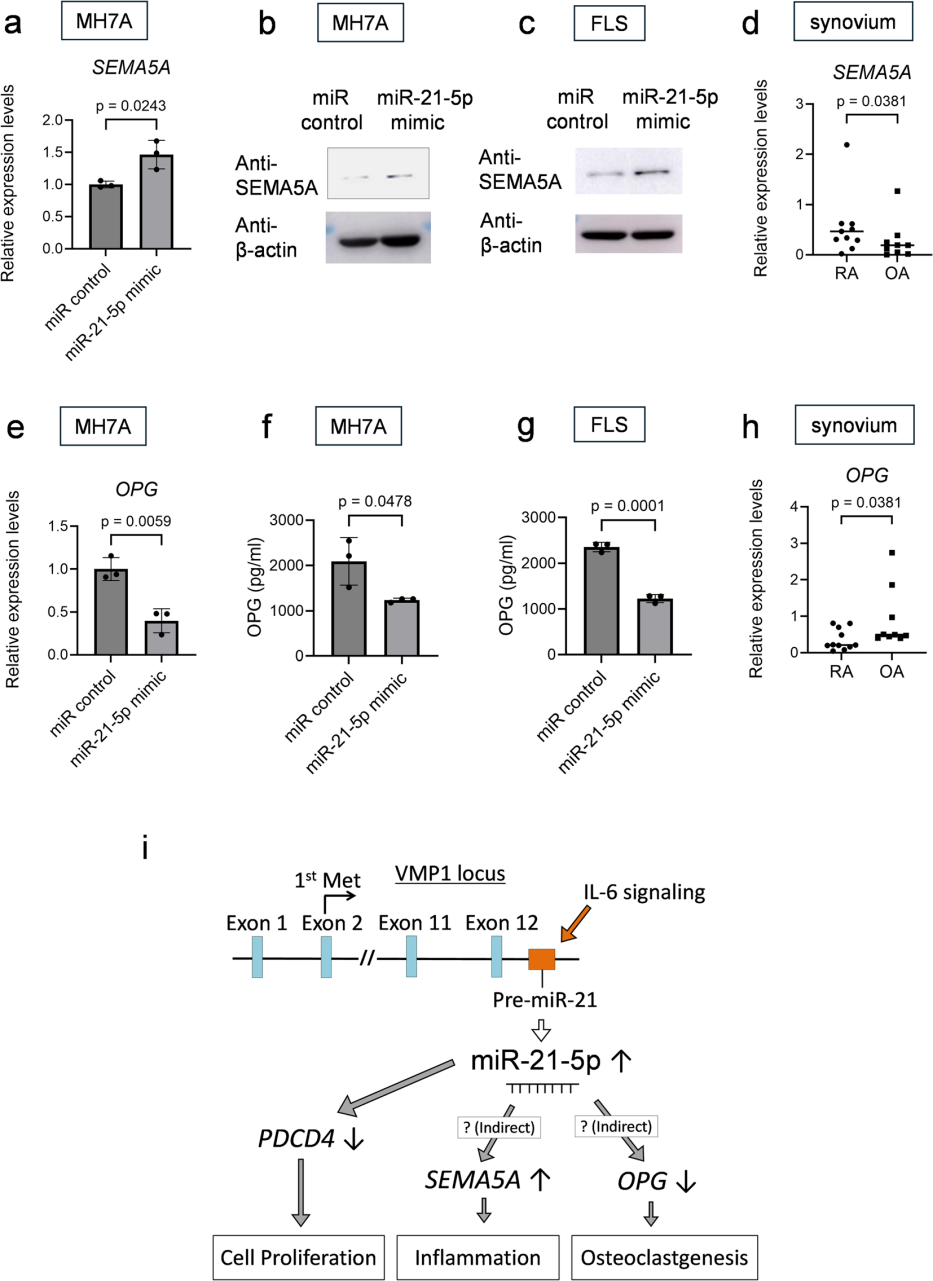
Hsa-miR-21-5p might indirectly influence synovial functions. (a, b, e, f) MH7A cells were transfected with miR-21-5p mimic or miR control. (c, g) Primary cultured FLS were transfected with miR-21-5p mimic or miR control. (**a**) RT-qPCR for *SEMA5A* expressions (*n* = 3). (**b**, **c**) SEMA5A proteins were detected by Western blotting. (d, h) Whole synovial tissues collected from patients with RA and OA. (**d**) RT-qPCR for SEMA5A expressions (RA; *n* = 11 vs. OA; *n* = 9). (**e**) RT-qPCR for *OPG* expressions (*n* = 3). (**f**, **g**) Supernatants of each cell after transfection were collected. Cytometric bead array (CBA) assay was performed to measure the OPG protein concentrations (*n* = 3). (**h**) RT-qPCR for *OPG* expressions (RA; *n* = 11 vs. OA; *n* = 9). (a, e, f, g) Data are presented as mean ± the standard error of the mean, t test. (d, h) Bars represent the mean value, and statistical analysis was performed using U-test. (**i**) The schema shows the latent function of hsa-miR-21-5p. MiR-21-5p decreases PDCD4 expression, thereby enhancing cell proliferation. It could also upregulate SEMA5A, which contributes to inflammation and enhances osteoclastogenesis through the reduction of OPG. SEMA5A, semaphorin 5A; OPG, osteoprotegerin; PDCD4, programmed cell death 4; VMP1, vacuole membrane protein 1.

Among the genes identified using conventional RNA-seq, indirect targets of hsa-miR-21-5p may also contribute to the pathogenesis of RA in FLS and rheumatoid synovial tissues. Notably, *semaphorin 5A* (*SEMA5A*) was included in the top 10 upregulated genes (Supplementary Fig. S6). SEMA5A is a biomarker for RA^24^, and soluble SEMA5A increases the proliferation of T lymphocytes and NK cells and induces the secretion of proinflammatory cytokines. Based on our data, the miR-21-5p mimic induced SEMA5A expression in MH7A cells and primary cultured FLS (Fig. 6a and b, and c). These data were supported by the expression levels of *SEMA5A* in synovial tissues using RT-qPCR; *SEMA5A* expression levels of RA were higher than those of OA (Fig. 6d).

*OPG* was also identified among the downregulated genes (Fig. 4b and c). *OPG*, a soluble decoy receptor for RANKL, is a naturally occurring inhibitor of the RANKL/RANK interaction that regulates osteoclastogenesis, and is expressed in the RA synovium^25,26^. In MH7A cells and primary cultured FLS, the miR-21-5p mimic reduced the *OPG* expression and concentrations in the culture supernatant (Fig. 6e and f, and g). Conversely, a miR-21-5p inhibitor upregulated *OPG* expression (Supplementary Fig. S7). The *OPG* expression levels in RA synovial tissues were lower than those in OA specimens (Fig. 6h). These data suggest that inflammatory conditions could lead to the upregulation of SEMA5A and downregulation of OPG, with the upregulation of hsa-miR-21-5p.

These above experiments revealed that hsa-miR-21-5p decreased the expression level of PDCD4 and increased the cell proliferation of FLS. In addition, it indirectly upregulated SEMA5A expression and downregulated OPG expression. These hsa-miR-21-5p systems in FLS may exacerbate the pathophysiology of rheumatoid synovitis, which is influenced by cell proliferation, inflammation, and promotion of osteoclastogenesis (Fig. 6i).

## Discussion

RA is characterized by chronic polyarthritis and systemic inflammation; therefore, miRNAs upregulated under inflammatory conditions may play a significant role in the pathogenesis of RA. Several miRNAs have been reported to regulate the key processes in RA-FLS, including migration, apoptosis, proliferation, and inflammation^27^. Although more than 2,000 miRNAs are registered in the database, no study has reported a comprehensive analysis to identify the abundantly expressed miRNAs on RA-FLS. Similarly, no previous studies have addressed the classification of miRNA coding locations in RA-FLS. Through the comprehensive small RNA-seq analysis of miRNA expression in RA-FLS, we reported intronic miRNAs as the most common category and identified the most abundantly expressed miRNAs in this population. Additionally, given that primary RA-FLS is cultured in a non-inflammatory environment during the preparation process, we aimed to clarify the differences between RA and OA or healthy controls by detecting cytokine-induced alterations of miRNAs expression levels. Among these intronic miRNAs, miR-21-5p was significantly upregulated by IL-6 stimulation and regulated independently of its host gene, *VMP1*. Thus, pri-miR-21-5p expression levels were higher in the RA synovium than those in the OA synovium, although the *VMP1* expression levels were similar.

In our study, the regulation and target genes of miR-21-5p in FLS were also investigated. Based on previous reports, IL-34 upregulates the miR-21-5p expression via STAT3 activation in RA-FLS, contributing to apoptosis resistance by modulating the ratio of B-cell lymphoma 2 to Bcl-2-associated X protein (Bcl-2/Bax ratio)^28^. Myostatin downregulates the miR-21-5p expression in MH7A cells^29^. Additionally, stimulation with lipopolysaccharide (LPS) in MH7A cells upregulates miR-21-5p^30^. These reports did not mention the expression levels of the host gene *VMP1*. Our data showed that IL-6 also induces miR-21-5p expression through STAT3 independent of VMP1.

Regarding miRNA target genes, miR-21-5p suppressed the Sucrose Nonfermentable 5 (SNF5) expression in MH7A cells and activated the nuclear factor kappa B (NF-κB) and phosphatase and tensin homolog/phosphatidylinositol 3-kinase/AKT serine/threonine kinase 1 (PTEN/PI3K/AKT) pathways, which are critical for exacerbating inflammation^30^. MiR-21-5p in TNFα-stimulated FLS suppressed the apoptosis and promoted cell invasion via the activation of PTEN/PI3K/AKT signaling by repressing PTEN as a target gene^31^. YOD1 deubiquitinase (YOD1) and yes-associated protein (YAP), which are key effectors of the Hippo signaling pathway, are direct targets of miR-21-5p in FLS^32^. We could not find any previous studies that used next-generation sequencing to analyze miR-21-5p targets in primary cultured FLS. Based on our data, conventional RNA-seq of FLS showed that miR-21-5p suppressed *PDCD4*, *RP2*, *DDAH1*, *TNFRSF11B (OPG)*, and *ID3*. In addition, both *PDCD4* and *RP2* sequences were included among the targets of hsa-miR-21-5p predicted using the TargetScan database. Therefore, PDCD4 and RP2 are the direct targets of hsa-miR-21-5p.

Many cancer studies have shown that *PDCD4* is a gene known to be associated with promoting apoptosis^33–36^. In glioblastoma-derived cell lines, the suppression of miR-21 restored the PDCD4 expression and promoted apoptosis^36^. Our data indicated that hsa-miR-21-5p also contributes to the FLS cell proliferation by inhibiting the PDCD4 expression as a direct target gene. As synovial tissue proliferation is an important component in the pathophysiology of RA synovitis, miR-21-5p may play an important role in RA. The transfected miR-21-5p mimic also suppressed RP2 expression, similar to PDCD4. However, this study did not demonstrate the involvement of RP2 in RA synovitis. Further analysis of RP2 will be required in the future.

In addition, several disease-related genes have been identified as candidates using conventional RNA-seq, although they are not expected to be direct targets. OPG, a soluble decoy receptor of RANKL, suppresses osteoclast progenitor cell differentiation and bone resorption^37^. In this study, the miR-21-5p mimic suppressed the OPG expression; therefore, miR-21-5p may affect bone erosion and joint destruction by downregulating the OPG expression. SEMA5A has been reported to be a biomarker for RA^24^. In Th1/Th17 cells, SEMA5A markedly promoted the secretion of proinflammatory cytokines, especially IL-17A and IL-22. In NK cells, SEMA5A significantly promotes the secretion of IL-6 and IL-8^24^. The effects of a fully human SEMA5A blocking monoclonal antibody (SYD12-12), evaluated in a collagen-induced arthritis mouse model, showed that SYD12-12 alleviated inflammatory cell infiltration, synovial hyperplasia, and bone destruction, and decreased serum levels of inflammatory cytokines, such as IL-1β, IL-6, TNF-α, and IL-17A^38^. The miR-21-5p mimic upregulated SEMA5A expression. Therefore, hsa-miR-21-5p could affect the inflammation of RA synovitis through SEMA5A upregulation, which leads to increased secretion of proinflammatory cytokines through T lymphocytes and NK cells. Similarly, in our study, miR-21-5p mimic slightly induced *IL1B* and *CXCL8* expressions in MH7A cells (Supplementary Fig. S10); however, the relationship between miR-21-5p, SEMA5A and these cytokines in FLS remains unclear. Whether miR-21-5p contributes significantly to synovial inflammation via SEMA5A should be further investigated.

## Conclusions

A comprehensive analysis using small RNA-seq revealed that some intronic miRNAs are abundantly expressed in FLS. Hsa-miR-21-5p upregulated by IL-6 promoted FLS proliferation in parallel with a decrease in PDCD4 expression. It also indirectly upregulated SEMA5A and downregulated OPG expression. Hsa-miR-21-5p in FLS may exacerbate the pathophysiology of rheumatoid synovitis.

## Methods

### Study design

This study was approved by the clinical ethics committees of Hiroshima University Hospital, Dohgo Spa Hospital, Ehime University Proteo-Science Center, and Graduate School of Medicine (approval number: E-668; approval date: 01/02/2017). All experiments were performed according to the approved guidelines. Synovial tissues were collected from patients with both OA and RA who fulfilled the classification criteria of the 1987 American College of Rheumatology^39^ and underwent total joint replacement after providing informed consent.

### Primary cultured and immortalized FLS preparation and culture

Primary cultured human FLS were isolated from the synovial tissue of patients with RA. Briefly, synovial tissues from total joint replacement lesions were minced and incubated with 1 mg/mL of collagenase/dispase (Roche, Mannheim, Germany) in phosphate-buffered saline (PBS) at 37 °C for 1 h, and subsequently filtered. For FLS preparation, the synovial cells were diluted and cultured. During culture, the supernatant was replaced frequently to dilute non-adherent cells. This is a necessary step to obtain a single adherent population of FLS, as freshly collected synovial tissues contain a variety of cells, including lymphocytes and macrophages. The adherent FLS were split at a 1:3 ratio under sub-confluent and passaged conditions. The FLS were cultured in Dulbecco’s Modified Eagle Medium (DMEM) (FUJIFILM Wako Pure Chemical Co., Osaka, Japan) supplemented with 10% fetal bovine serum (FBS) (Gibco, Waltham, MA, USA) and penicillin/streptomycin (FUJIFILM Wako Pure Chemical Co.). The FLS were used for experiments at passages 3–6. For cytokine stimulation using recombinant human TNF-α, IL-1β, IL-6, sIL-6R, and IL-17 (Biolegend, San Diego, CA, USA), the FLS were starved in DMEM with 0.1% FBS for 24 h before the addition of recombinant proteins.

The immortalized FLS from patients with RA (MH7A cells) were obtained from KISSEI Pharmaceutical Co., Ltd. (Matsumoto, Japan). The MH7A cells were cultured in Roswell Park Memorial Institute (RPMI) 1640 medium (FUJIFILM Wako Pure Chemical Co.) supplemented with 10% FBS and penicillin/streptomycin.

### RNA extraction and RT-qPCR

Total RNA was extracted and purified from whole synovial tissues and cultured cells using TRIzol reagent (Life Technologies, Carlsbad, CA, USA). For miRNA purification and next-generation sequencing preparation, a Direct-zol RNA kit (Zymo Research, Irvine, CA, USA) with TRIzol reagent was used. For cDNA synthesis from mRNA, PrimeScript RT Reagent Kit with gDNA Eraser (Takara Bio, Kusatsu, JAPAN) was used. RT-qPCR was performed with Brilliant II SYBR Green QPCR Master Mix (Agilent, Santa Clara, CA, USA) using the CFX Connect Real-Time PCR Detection System (Bio-Rad Laboratories, Hercules, CA, USA). The primers used in this study are listed in Table 1. The gene expression levels were normalized to the housekeeping gene, *glyceraldehyde 3-phosphate dehydrogenase* (*GAPDH*). For cDNA synthesis from miRNA and RT-qPCR, TaqMan MicroRNA Assay kits (Applied Biosystems, Waltham, MA, USA) were used according to the manufacturer’s instructions. The TaqMan probes used are listed in Table 2. The data were normalized to the endogenous reference gene, U6 snRNA.

**Table 1:** List of RT-qPCR primer sequences.

Gene symbol	Sequence (5′-3′) for forward	Sequence (5′-3′) for reverse
*AOPEP*	GTGTGGAGAGGTTCCTTCAGG	GACCTATCCATCTGCTCCTTG
*CCL2*	AGACTAACCCAGAAACATCC	ATTGATTGCATCTGGCTG
*COPZ1*	GCTGTTCTGAACTGTCTCTTCG	CATCCACAGCCAAGAACAGCC
*CXCL8*	GTTTTTGAAGAGGGCTGAG	TTTGCTTGAAGTTTCACTGG
*CXCL10*	AAAGCAGTTAGCAAGGAAAG	TCATTGGTCACCTTTTAGTG
*EBI3*	TAACAGAGCATCATCAAG	TTCAGTGAGAAGATCTCTGG
*EVL*	GACCTCAAAGTCCGATGCCAA	GTCTGACTGGGAGGCTGCTTT
*FOXO1*	TACGAGTGGATGGTCAAGAGC	CAGTTCCTTCATTCTGCACACG
*FOXO3*	CTACGAGTGGATGGTGCGTTG	TCTTGCCAGTTCCCTCATTCTG
*GAPDH*	AAGGTCATCCCAGAGCTGAA	CTGCTTCACCACCTTCTTGA
*HMGCR*	ACGTGAACCTATGCTGGTCAG	GTATCTGTTTCAGCCACTAAGG
*IL10*	GCCTTTAATAAGCTCCAAGAG	ATCTTCATTGTCATGTAGGC
*IL1B*	CTAAACAGATGAAGTGCTCC	GGTCATTCTCCTGGAAGG
*IL6*	GCAGAAAAAGGCAAAGAATC	CTACATTTGCCGAAGAGC
*MCM7*	CCAAGTCTCAGCTCCTGTCAT	CTCTAAGGTCAGTTCTCCACTC
*NDUFAF3*	CTTTTCCCTCTTCTGGTTGCTG	TTGAAGGTGGCACAGGCATTG
*NSRP1*	AAGAGGGCTGCTGCACTGGAA	GCTTCACGAAAGCTGCATTTAG
*OPG*,* TNFRSF11B*	ATAGATGTTACCCTGTGTGAG	AAGACACTAAGCCAGTTAGG
*PDCD4*	CTGTGCCAACCAGTCCAAAGG	CTCCACATCATACACCTGTCC
*PPARG*	GCCTGCGAAAGCCTTTTGGTG	GCTTCACATTCAGCAAACCTGG
*RP2*	TCAGAGACAGAAGAGCAGCGA	GACACTTCCTTTGTCTGAACTAG
*RUNX1*	CACCTACCACAGAGCCATCAA	TCACTGAGCCGCTCGGAAAAG
*SEMA5A*	CTGAAGAGGTGCCAGTTCTAC	CAGAGATGCTCTGTTCCCACT
*SLC7A11*	CCTGCTTTGGCTCCATGAACG	GAGGAGTGTGCTTGCGGACAT
*SMAD2*	GGTTTTGAAGCCGTCTATCAGC	CAACCACTGTAGAGGTCCATTC
*SMC4*	CCCAAGTAGCAATCAAGACTGC	CTCTGCTGTTAGGTCATCCAC
*TNF*	AGGCAGTGAGATCATCTTC	TTATCTCTCAGCTCCACG
*VEGFC*	CCAATCACACTTCCTGCCGAT	GGTCTTGTTCGCTGCCTGACA
*VMP1* (exon5)	TGGAACAGGGCTGCACACCTT	CAGGATAGGGTGGTTCGGGAA
*VMP1* (exon12)	ACACCACAGGGAGAAAACTGGT	CTGCTGGATTCGTTTGGCATAAC
*VMP1* (pre-miR-21)	TGTCGGGTAGCTTATCAGAC	TGTCAGACAGCCCATCGACT
*VMP1* (pri-miR-21)	AGTCGTGGTTCATCTCTTTCACC	GCCAATGAATCCCAAAGATTTGG
*WWP2*	GTGCGATACTTTGTGGACCAC	ATACTTCCACCGAAAACTGCGG

**Table 2:** List of TaqMan MicroRNA probes.

Gene symbol	Probe ID (from Applied Biosystems)
hsa-miR-15b-5p	000390 (Taqman MicroRNA Assays)
hsa-miR-21-5p	000397 (Taqman MicroRNA Assays)
hsa-miR-21-3p	002438 (Taqman MicroRNA Assays)
hsa-miR-27b-3p	000409 (Taqman MicroRNA Assays)
hsa-miR-93-5p	001090 (Taqman MicroRNA Assays)
hsa-miR-140-3p	002234 (Taqman MicroRNA Assays)
hsa-miR-148b-3p	000471 (Taqman MicroRNA Assays)
hsa-miR-191-5p	002299 (Taqman MicroRNA Assays)
hsa-miR-342-3p	002260 (Taqman MicroRNA Assays)
hsa-miR-423-5p	002340 (Taqman MicroRNA Assays)
U6 snRNA	001973 (Taqman MicroRNA Assays)

### Next-generation sequencing (small RNA-seq)

Small RNA sequence library preparation, sequencing, mapping, and gene expression analyses were performed using DNAFORM Inc. (Yokohama, Kanagawa, Japan). The quality and quantity of the extracted miRNAs were assessed using the QuantiFluor RNA System (Promega, Madison, WI, USA) and Agilent Small RNA kit of the BioAnalyzer 2100 System (Agilent Technologies) according to the manufacturer’s instructions. A small RNA-seq library was prepared using the QIAseq miRNA Library Kit (Qiagen, Venlo, Netherlands), following the manufacturer’s instructions. In brief, a pre-adenylated DNA adapter was ligated to the 3’ ends of all miRNAs. An RNA adapter was ligated to the 5’ end of mature miRNAs. Subsequently, reverse transcription (RT) was performed using RT primers containing Unique Molecular Indices. Finally, the library was amplified using the sample-specific index primers. The libraries were sequenced on a NextSeq 500 sequencer (Illumina, San Diego, CA, USA) to generate 75 nt single reads. Duplicate reads were removed from the raw reads using Seqkit (ver. 0.10.1, https://bioinf.shenwei.me/seqkit/), and the adapter sequence was trimmed using Cutadapt (ver. 1.16, https://pypi.org/project/cutadapt/). The trimmed reads were mapped to the human genome (the genome assemblies used were GRCh38.p13) using STAR (ver. 2.7.2b, https://github.com/alexdobin/STAR/releases). The reads mapped to the miRNA genes were quantified using featureCounts in the Subread package (ver. 1.6.1, https://subread.sourceforge.net/). The miRNAs were classified as intergenic, intronic, exonic, or others (unclassifiable) using the UCSC genome browser (human, hg38, https://genome-asia.ucsc.edu/index.html, accessed on April/13/2024).

### SiRNA and MiRNA mimic transfection into primary cultured FLS and MH7A cells

The siRNA against STAT3 (FlexiTube siRNA, SI02662898), siRNA against RP2 (FlexiTube siRNA, SI00093205) and siRNA negative control (Allstar Negative Control siRNA) were purchased from Qiagen. The miRNA mimics, hsa-miR-21-5p and hsa-miR-27b-3p, and Synthetic Tough Decoy (S-TuD) against miR-21-5p were purchased from Ajinomoto Bio-Pharma (Osaka, Japan). MiR control (MISSION miRNA negative control) was purchased from Sigma-Aldrich (St. Louis, MO, USA). For these small RNA transfections, primary cultured FLS and MH7A cells were treated using Lipofectamine RNAiMAX (Invitrogen, Carlsbad, CA, USA) according to the manufacturer’s protocol. Harvested cells and supernatants were used in subsequent experiments.

### Next-generation sequencing (conventional RNA-seq)

Conventional RNA sequence library preparation, sequencing, mapping, and gene expression analyses were performed using DNAFORM Inc. Total RNA quality was assessed using a BioAnalyzer (Agilent Technologies) to ensure that the RNA integrity number was greater than 7.0. Double-stranded cDNA libraries (RNA-seq libraries) were prepared using the SMART-Seq Stranded Kit (Clontech, Kusatsu, JAPAN, Cat. #634442) and DNBSEQ MGIEasy Universal Library Conversion Kit (MGI Tech, Shenzhen, China), according to the manufacturer’s instructions. The RNA-seq libraries were sequenced using paired-end reads (150nt of read1 and read2) on a DNBSEQ-G400RS instrument (MGI Technology). The raw reads obtained were trimmed and quality-filtered using Trim Galore! (version 0.6.7, https://github.com/FelixKrueger/TrimGalore), Trimmomatic (version 0.39, http://www.usadellab.org/cms/?page=trimmomatic), and Cutadapt (version 3.7, https://pypi.org/project/cutadapt/). The trimmed reads were mapped to the human GRCh38 genome using the STAR software (version 2.7.10a, https://github.com/alexdobin/STAR/releases). The reads on the annotated genes were counted using featureCounts (version 2.0.1, https://subread.sourceforge.net/featureCounts.html). The transcripts per million (TPM) and the fragments per kilobase of exon per million reads mapped (FPKM) values were calculated from the mapped reads by normalizing them to the total counts and transcripts. The genes with read counts < 5 were excluded and normalized to those of housekeeping genes. The relative expression values were calculated using the following formula: (1) Log2[(the gene read count of each sample)/(total average count of the gene)] and (2) the difference between the mean value of (1) in miR-21-5p mimic-transfected cells and that in miR control-transfected cells. False discovery rate (FDR) was controlled using the two-stage linear step-up procedure of the Benjamini, Krieger, and Yekutieli method to determine an optimal threshold for statistical significance. The upregulated and downregulated genes were identified based on larger fold-changes.

### Western blotting

Before sample preparation, MH7A cells and FLS were washed with PBS. The cells were homogenized in 2% sodium dodecyl sulfate sample buffer using BioMasher II (Nippi Corporation, Tokyo, Japan). Subsequently, the samples were centrifuged at 15,000 × g for 5 min at 20–25 °C, and the supernatant was collected. The lysates were electrophoresed on SuperSepAce 5–12% precast gels (FUJIFILM Wako Pure Chemical Co.) and transferred to polyvinylidene fluoride (PVDF) membranes. The membranes were probed with the following primary antibodies: anti- PDCD4 antibody (sheep polyclonal, R&D systems, Minneapolis, MN, USA), anti-SEMA5A antibody (rabbit polyclonal, Genetex, Irvine, CA, USA), and anti-human β-actin antibody (mouse monoclonal, clone AC-15, Sigma-Aldrich). Subsequently, the cells were treated with horseradish peroxidase (HRP)-conjugated secondary antibodies (host: donkey; Jackson ImmunoResearch, West Grove, PA, USA). The HRP activity was detected using the Image Quant LAS 500 system (Cytiva, Tokyo, Japan) with ECL Prime reagent (Cytiva).

### Cytometric bead array assay

The culture supernatants of MH7A cells and FLS treated with either miR-21-5p mimic or miR negative control for 48 h were harvested for sample preparation as described above. The OPG levels were measured using the LEGENDplex system (BioLegend) according to the manufacturer’s protocol.

### IHC staining for formalin-fixed paraffin-embedded (FFPE) synovial tissues

The sections were obtained from FFPE synovial tissues. After deparaffinization and antigen retrieval in Tris-EDTA buffer (pH 9.0), the specimens were first incubated with primary antibodies (anti-RP2, rabbit polyclonal, Proteintech (Rosemont, IL, USA), anti-PDCD4, sheep polyclonal, R&D Systems), and then with biotin-conjugated secondary antibodies purchased from Jackson ImmunoResearch. The slides were then treated with alkaline phosphatase (AP)-conjugated streptavidin (Jackson ImmunoResearch) and visualized using ImmPACT Vector Red Alkaline Phosphatase Substrate (Vector Laboratories, Burlingame, CA, USA). The slides were counterstained with hematoxylin solution.

### IVT mRNA transfection into MH7A cells

A plasmid encoding HA-tagged full-length PDCD4 (NM_014456.5) or an empty control vector (mock) based on the pcDNA3-A(124) vector was constructed, and in vitro transcribed (IVT) mRNA was prepared as previously described^40^. The insert fragment of the HA-tagged PDCD4 open reading frame was purchased from GenScript (Piscataway, NJ, USA). The IVT mRNA was transfected into cells using Lipofectamine Messenger MAX (Invitrogen), according to the manufacturer’s instructions. Briefly, IVT mRNA was blended with the Lipofectamine reagent in a ratio of 1:3 (pmol mRNA: µL Lipofectamine) in serum-free RPMI, and subsequently incubated for 20 min at 25 °C for complex formation. After transfection, the cells were harvested for subsequent experiments.

### Cell viability assay using calcein-AM

MH7A cells (5.0 × 10^3^ cells/well) were seeded in 96-well plates using 10% FBS supplemented RPMI medium. The cells were cultured in each well for 24 h and then transfected with either miR control, miR-21-5p mimic, mock IVT mRNA, or PDCD4 IVT mRNA, as described above. The cell viability was assessed using calcein-AM (Cell Counting Kit-F; Dojindo Laboratories, Kumamoto, Japan) at 0, 24, 72, and 96 h after transfection, according to the manufacturer’s protocol. Calcein fluorescence was quantified using a SpectraMax iD3 system (Molecular Devices, San Jose, CA, USA) at excitation and emission wavelengths of 485 and 525 nm, respectively.

### Statistical analysis

The statistical differences between pairs of groups were assessed using an unpaired t-test (Student’s t-test) or a non-parametric Mann–Whitney U-test. To complement the hypothesis testing with estimation, we also calculated the effect size (Cliff’s delta for Mann–Whitney U-test) and interpreted the values greater than 0.33 as indicating a moderate or larger effect. The differences between three or five groups were determined using the Kruskal–Wallis test, followed by the Dunn test. Data processing and analyses were performed using GraphPad Prism 10 (version 10.4.0, https://www.graphpad.com/; GraphPad Software Inc., La Jolla, CA, USA). Data are presented as the mean ± standard deviation or mean ± standard error of the mean.

## Electronic supplementary material

Below is the link to the electronic supplementary material.

Supplementary Material 1

## Data Availability

The datasets analyzed in this study are available from the corresponding author upon request. Some data are not available because they contain information that could compromise the privacy of the participants. The small RNA-seq and conventional RNA-seq raw data are available in the NCBI Sequence Read Archive with BioProject ID PRJNA1158331.
