# Supplementary material for: Hsa-miR-21-5p is induced by interleukin-6 and affects multiple pathogenic factors associated with fibroblast-like synoviocytes in rheumatoid arthritis
Source: Sci Rep. 2025 Jun 3;15:19416. doi: 10.1038/s41598-025-02840-z (PMC12134285; doi:10.1038/s41598-025-02840-z)

Supplementary Figures

Hsa-miR-21-5p is induced by interleukin-6 and affects multiple pathogenic factors associated with fibroblast-like synoviocytes in rheumatoid arthritis

Araki K and Mokuda S, et al.

- Supplementary Figure S1 – S10
- Uncropped blotting data

Supplementary Figure S1.
Highly expressed intronic miRNA and their host genes in FLS

	Read counts, average	miRNA	Host gene
1	991,573	hsa-miR-191-5p	*NDUFAF3; NADH:Ubiquinone Oxidoreductase Complex Assembly Factor 3*
2	712,346	hsa-miR-148b-3p	*COPZ1; COPI Coat Complex Subunit Zeta 1*
3	375,792	hsa-miR-93-5p	*MCM7; Minichromosome Maintenance Complex Component 7*
4	359,327	hsa-miR-140-3p	*WWP2; WW Domain Containing Protein 2*
5	345,487	hsa-miR-423-5p	*NSRP1; Nuclear Speckle Splicing Regulatory Protein 1*
6	345,437	hsa-miR-27b-3p	*AOPEP; Aminopeptidase O*
7	290,548	hsa-miR-15b-5p	*SMC4; Structural Maintenance of Chromosomes 4*
8	232,426	hsa-miR-342-3p	*EVL; Enah/Vasp-Like*
9	231,192	hsa-miR-21-3p	*VMP1; Vacuole Membrane Protein 1*
10	183,879	hsa-miR-21-5p	*VMP1; Vacuole Membrane Protein 1*
11	151,443	hsa-miR-615-3p	*HOXC5; Homobox C5*
12	150,669	hsa-miR-328-3p	*ELMO3; Engulfment And Cell Motility 3*
13	142,538	hsa-miR-423-3p	*NSRP1; Nuclear Speckle Splicing Regulatory Protein 1*
14	111,319	hsa-miR-532-5p	*CLCN5; Chloride Voltage-Gated Channel 5*
15	105,769	hsa-miR-23b-3p	*AOPEP; Aminopeptidase O*
16	93,457	hsa-miR-30e-5p	*NFYC; Nuclear Transcription Factor Y Subunit Gamma*
17	87,026	hsa-miR-744-5p	*MAP2K4; Mitogen-Activated Protein Kinase Kinase 4*
18	64,407	hsa-miR-25-3p	*MCM7; Minichromosome Maintenance Complex Component 7*
19	57,198	hsa-miR-425-5p	*DALRD3; DALR Anticodon Binding Domain Containing 3*
20	55,620	hsa-miR-361-3p	*CHM; CHM Rab Escort Protein*

The average read counts were obtained from the analyzed small RNA-seq.

Supplementary Figure S2. The relationship between hsa-miR-27b-3p and pathogenic factors associated with RA.

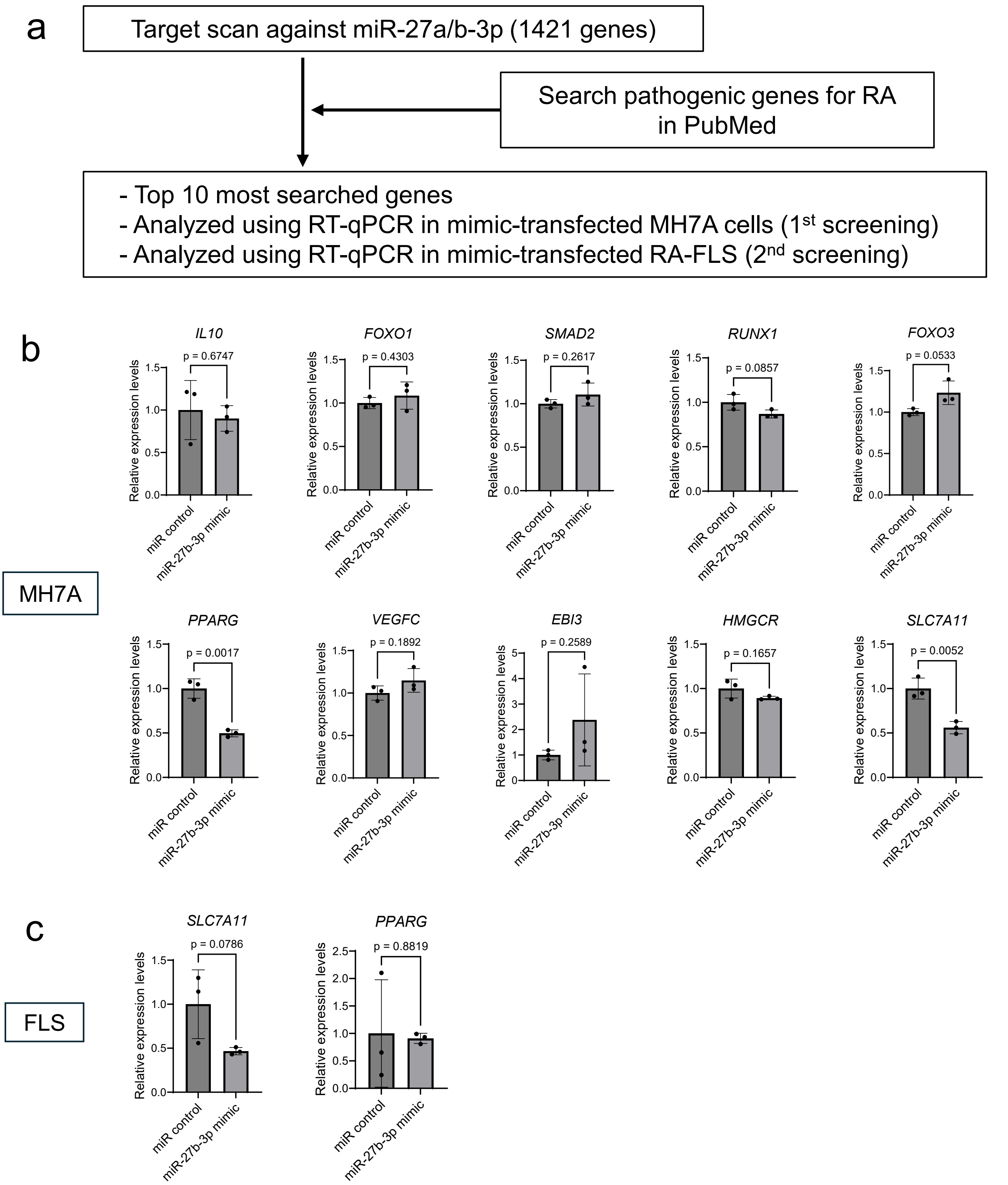

(a) Flow diagram illustrating the analysis of miR-27b-3p. (b, c) Cells were transfected with either miR-27b-3p mimic or miR negative control for 48 h. All expression levels were measured using RT-qPCR, and statistical analyses were performed using t-test. Data represent the mean ± the standard error of the mean. (b) MH7A cells (n = 3); (c) RA-FLS (n = 3, from individual subject). EBI3, Epstein-Barr virus induced gene 3; FOXO1, forkhead box protein O1; FOXO3, forkhead box protein O3; HMGCR, 3-hydroxy-3-methylglutaryl-CoA reductase; IL10, interleukin 10; PPARG, peroxisome proliferator activated receptor gamma; RUNX1, runt-related transcription factor 1; SLC7A11, solute carrier family 7 member 11; SMAD2, SMAD family member 2; VEGFC, vascular endothelial growth factor C.

Supplementary Figure S3.
Host gene expression levels corresponding to top 10 intronic miRNAs in FLS

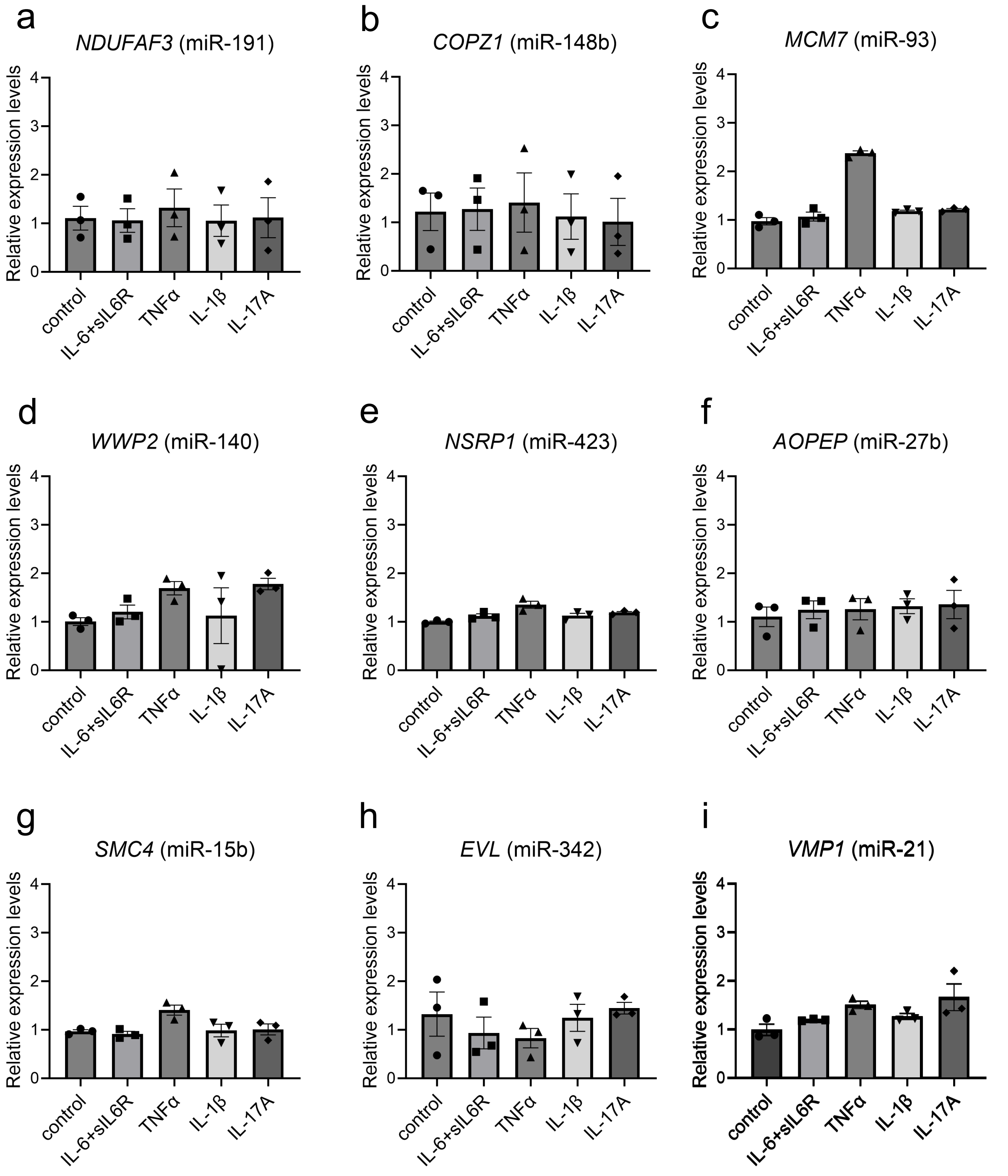

Fibroblast-like synoviocytes (FLS) collected from patients with rheumatoid arthritis (RA) were stimulated with TNFα (100 ng/mL), IL-1β (10 ng/mL), IL-6 (100 ng/mL) with 100 ng/mL of sIL-6R, and IL-17A (100 ng/mL) for 24 h. (a) *NDUFAF3*; (b) *COPZ1*; (c) *MCM7*; (d) *WWP2*; (e) *NSRP1*; (f) *AOPEP*; (g) *SMC4*; (h) *EVL*; and (i) *VMP1*. All expression levels were measured using RT–qPCR, and statistical analyses were performed using Dunn's test. Data represent the mean ± the standard error of the mean (n = 3, from each individual subject).

Supplementary Figure S4.
PCA analysis for conventional RNA-seq in miR-21-5p mimic-transfected FLS

a

TPM method

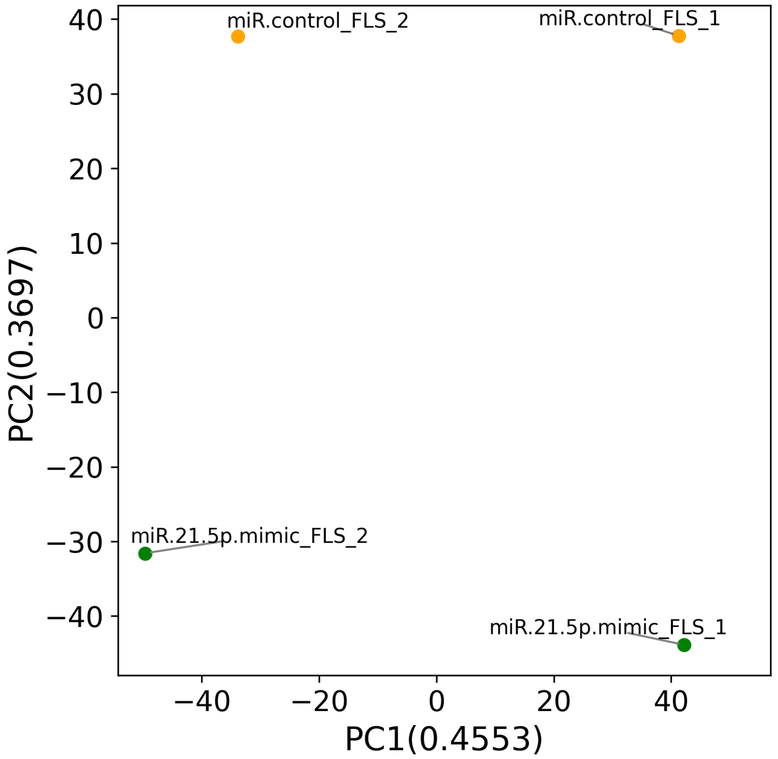

b

FPKM method

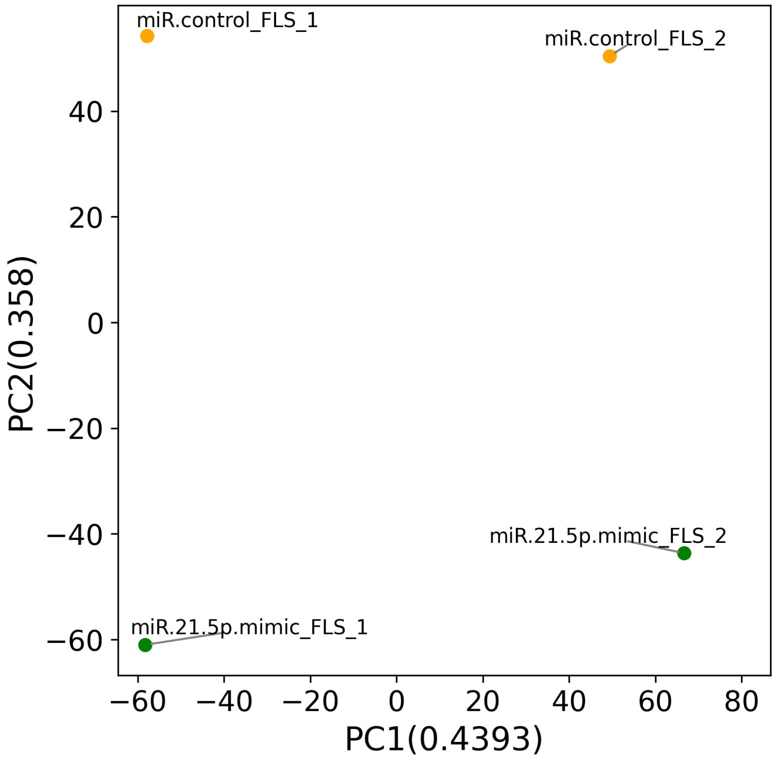

RNA from miR-21-5p mimic or miR negative control-transfected FLS was collected and analyzed using next-generation sequencing analysis (conventional RNA-seq) (n = 2, from each individual subject). These data display the result of principal component analysis (PCA). (a) TPM method. (b) FPKM method.

Supplementary Figure S5.
Result of BAM file mapping for conventional RNA-seq in miR-21-5p mimic-transfected FLS

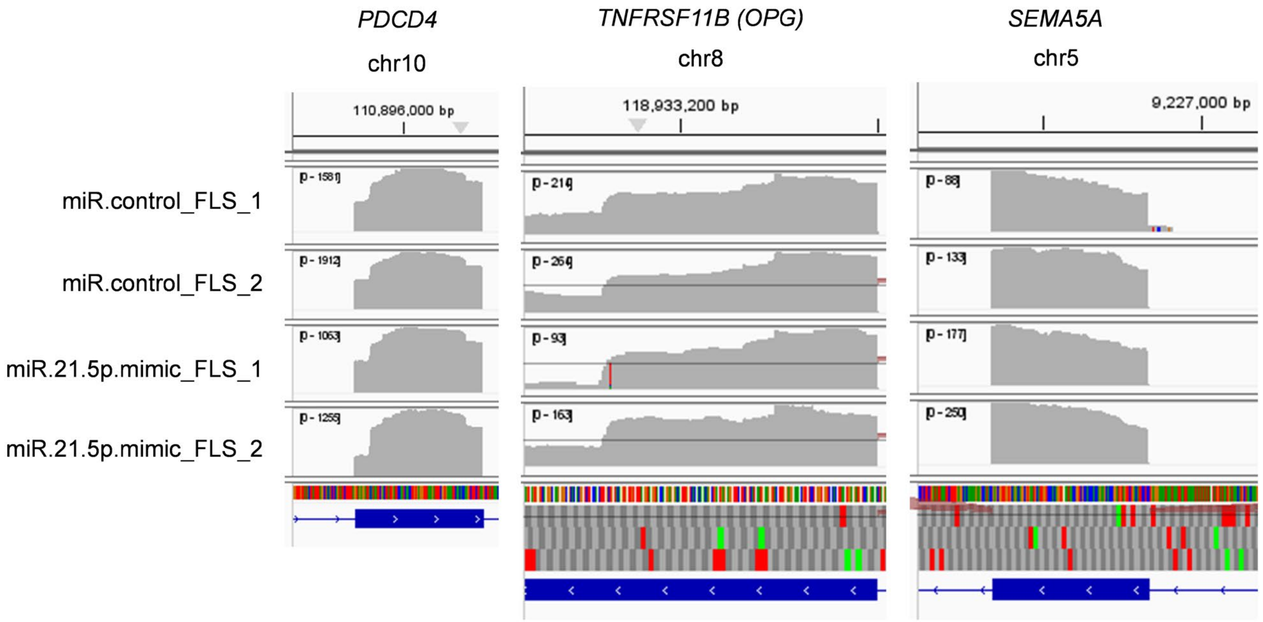

RNA from miR-21-5p mimic or miR negative control-transfected FLS was collected and analyzed using next-generation sequencing analysis (conventional RNA-seq) (n = 2, from each individual subject). These data display the result of BAM file mapping. The data were processed using the IGV (Integrative Genomics Viewer) software.

Supplementary Figure S6. Extra data for conventional RNA-seq

a TPM method

TOP 10 up-regulated		
Gene name (symbol)	Log fold change (miR-21-5p − control)	−log10 (p-value)
GANAB	1.69	1.49
HDDC2	1.49	2.44
ABRACL	1.42	2.02
LSM4	1.34	2.04
NTN4	1.25	2.29
ZMAT3	1.18	1.69
GFPT2	1.15	1.34
SARAF	1.14	2.10
RBPJ	1.12	2.23
SEMA5A	1.10	2.04

b FPKM method

TOP 10 up-regulated		
Gene name (symbol)	Log fold change (miR-21-5p − control)	−log10 (p-value)
ABRACL	1.51	2.30
LSM4	1.33	2.09
SEMA5A	1.28	1.98
IFIT2	1.25	1.36
ZMAT3	1.22	1.83
NTN4	1.22	2.23
SMIM14	1.21	1.40
GFPT2	1.14	1.78
SARAF	1.11	2.10
RBPJ	1.11	2.25

Top 10 upregulated genes from the RNA-seq dataset. Abbreviations are described in Supplementary Figure 7. (a) TPM method; (b) FPKM method. ABRACL, ABRA C-terminal Like; GANAB, glucosidase II alpha subunit; GFPT2, glutamine-fructose-6-phosphate transaminase 2; HDDC2, HD domain-containing protein 2; IFIT2, interferon induced protein with tetratricopeptide repeats 2; LSM4, LSM4 homolog; NTN4, netrin 4; RBPJ, recombination signal binding protein for immunoglobulin kappa J region; SARAF, store-operated calcium entry associated regulatory factor; SEMA5A, semaphorin 5A; SMIM14, small integral membrane protein 14; ZMAT3, zinc finger matrin-type 3.

Supplementary Figure S7. miR-21-5p inhibitor upregulates both *PDCD4* and *OPG* expressions

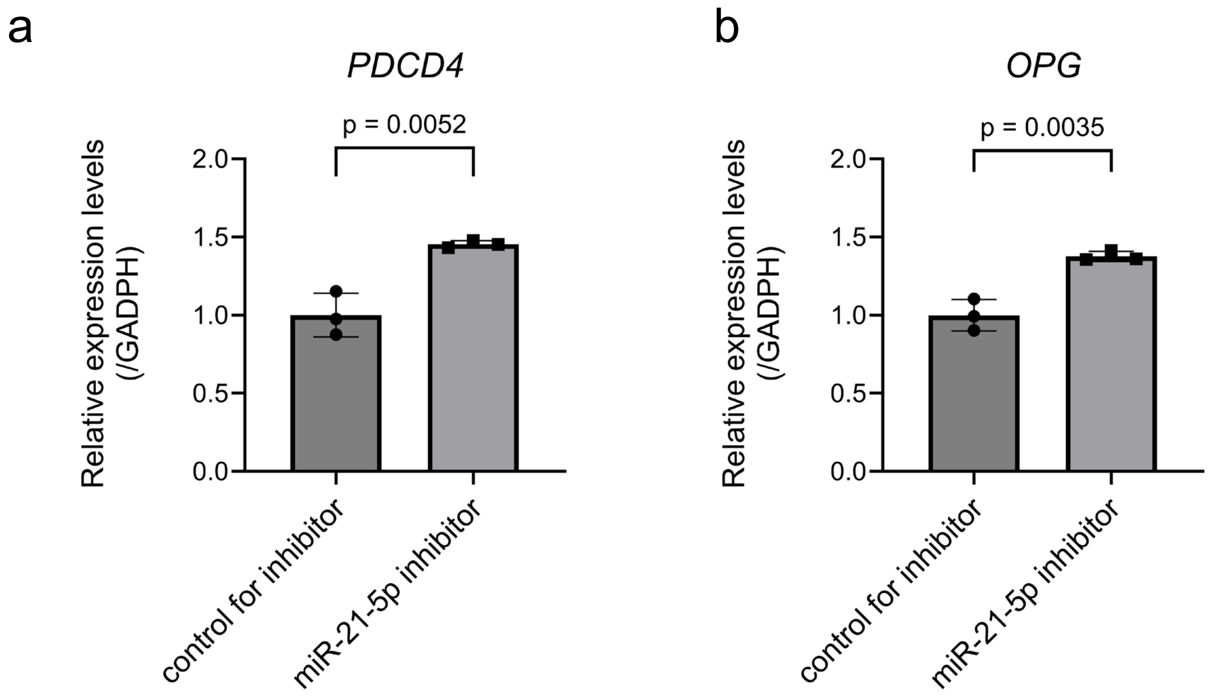

(a-b) MH7A cells were transfected with either miR-21-5p inhibitor (Synthetic Tough Decoy [S-TuD] against miR-21-5p) or S-TuD negative control for 48 h. (a) *PDCD4* expression; (b) *OPG* expressionAll expression levels were measured using RT-qPCR, and statistical analyses were performed using t-test. Data represent the mean ± the standard error of the mean (n = 3).

Supplementary Figure S8. Immunohistochemistry staining for RP2

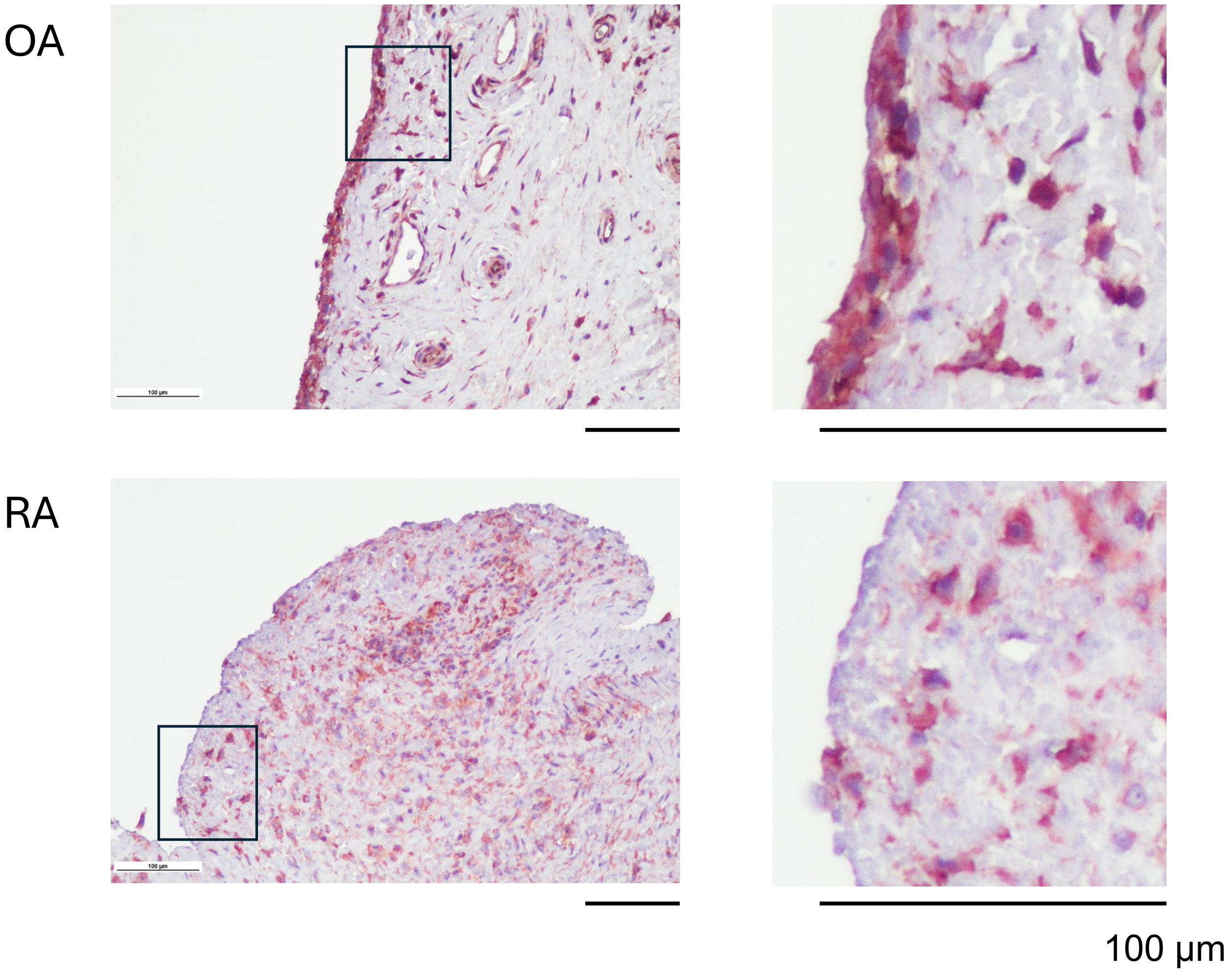

Formalin-fixed paraffin-embedded (FFPE) synovial tissues harvested from patients with RA and OA were stained with anti-RP2 antibody. RP2 expression in the synovial tissues of patients with OA was higher than that of RA tissues. Representative images are shown. Red, RP2; purple, hematoxylin; black scale bar = 100 µm. RP2, retinitis pigmentosa 2.

Supplementary Figure S9. Effect of siRNA against RP2 on FLS.

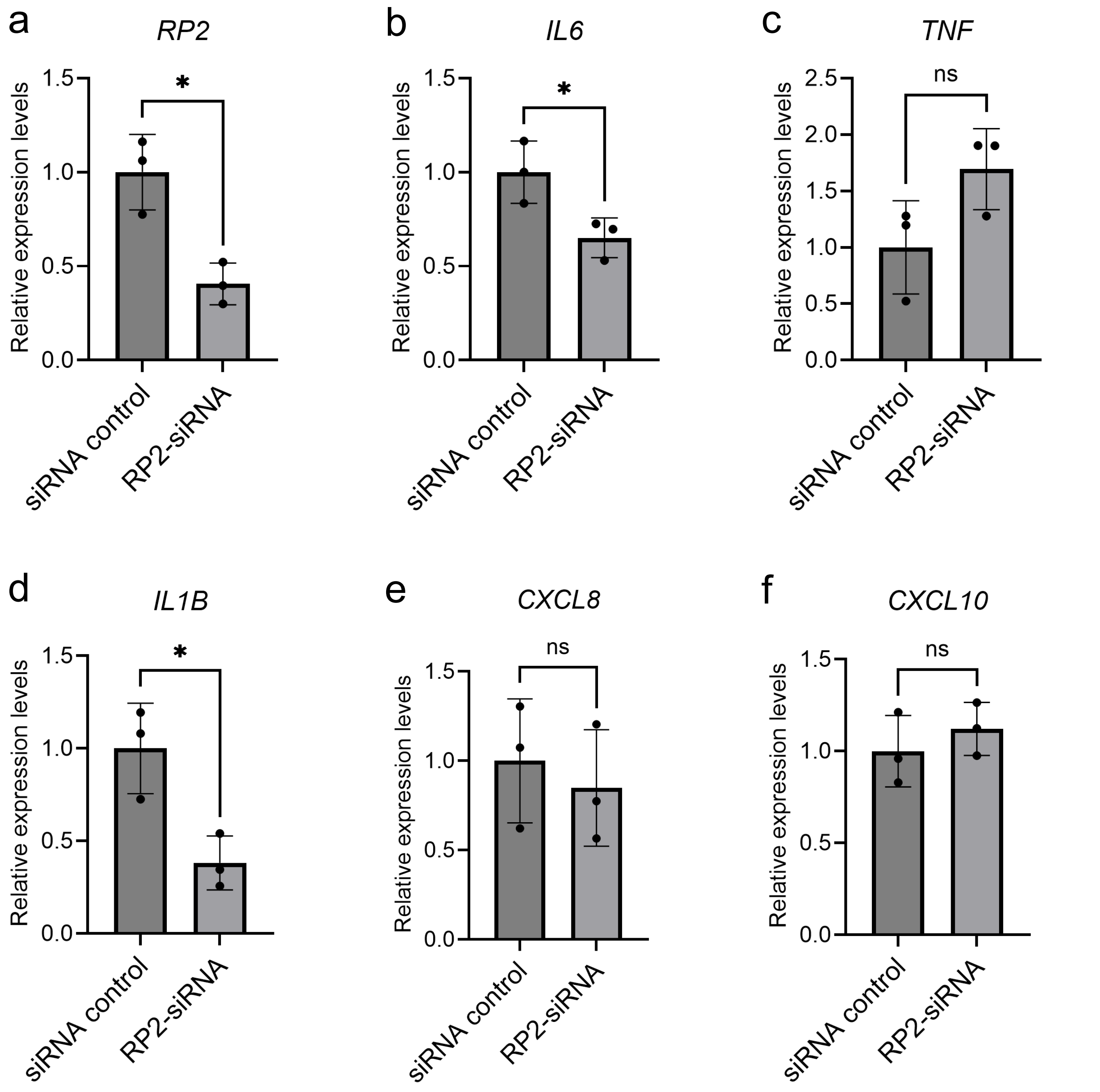

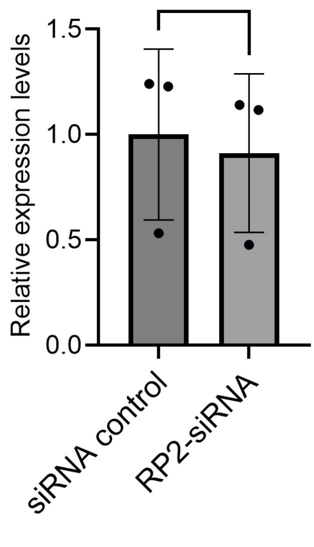

(a-g) MH7A cells were transfected with either siRNA against RP2 or siRNA negative control for 48 h. (a) *RP2* expression; (b) *IL6* expression; (c) *TNF* expression; (d) *IL1B* expression; (e) *CXCL8* expression; (f) *CXCL10* expression; (g) *CCL2* expression. All expression levels were measured using RT-qPCR, and statistical analyses were performed using t-test (*p<0.05). Data represent the mean ± the standard error of the mean (n = 3).

RP2, retinitis pigmentosa 2; IL6, interleukin-6; TNF, tumor necrosis factor; IL1B, interleukin-1β; CXCL8, C-X-C motif chemokine ligand 8; CXCL10, C-X-C motif chemokine ligand 10; CCL2, C-C motif chemokine ligand 2.

Supplementary Figure S10. miR-21-5p mimic slightly induces *IL1B* and *CXCL8* expressions

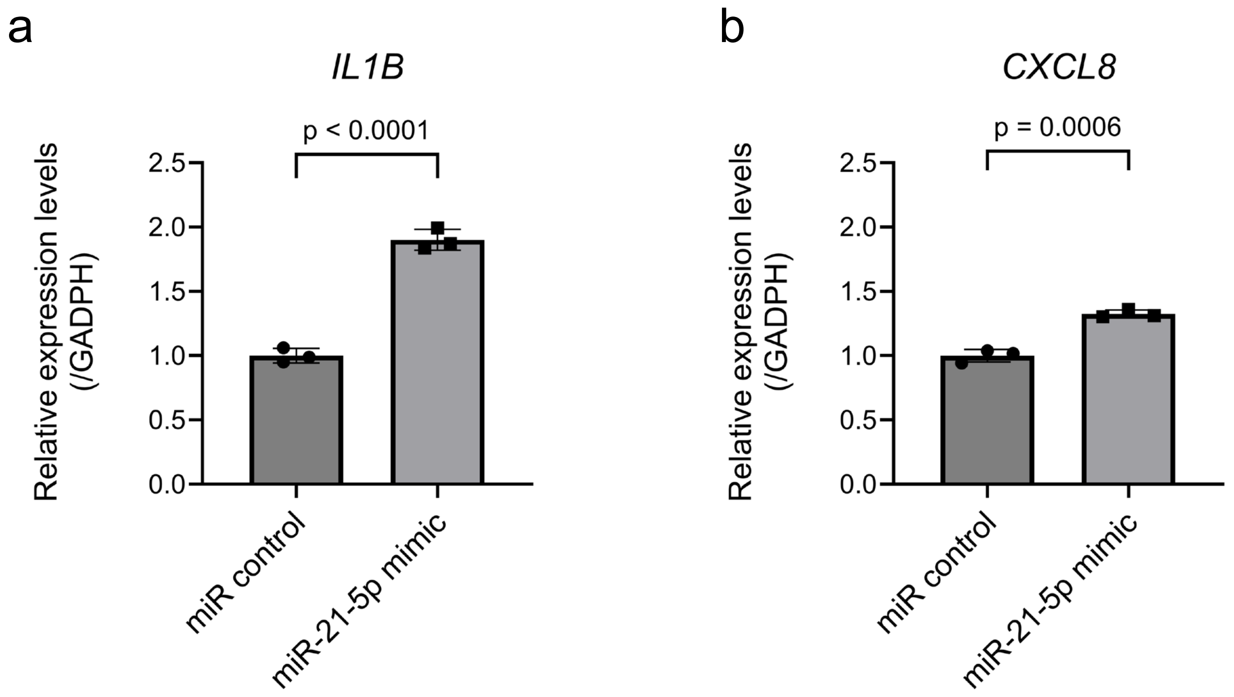

(a-b) MH7A cells were transfected with either miR-21-5p mimic or miR negative control for 48 h. (a) *IL1B* expression; (b) *CXCL8* expression. All expression levels were measured using RT-qPCR, and statistical analyses were performed using t-test. Data represent the mean ± the standard error of the mean (n = 3).

Additional Data (Uncropped images for blotting)

Figure 5b

Anti-PDCD4

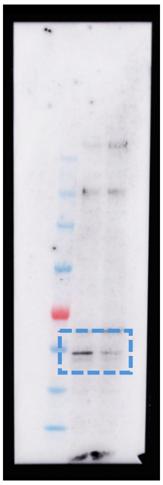

Anti-β-actin

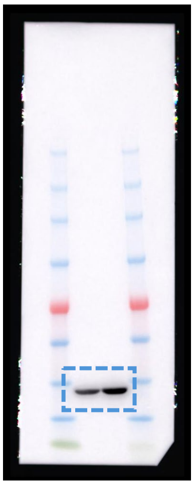

Figure 5f

Anti-PDCD4

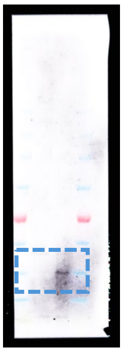

Anti-β-actin

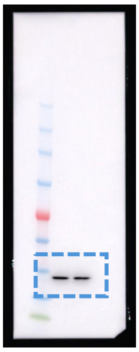

Figure 6b

Anti-SEMA5A

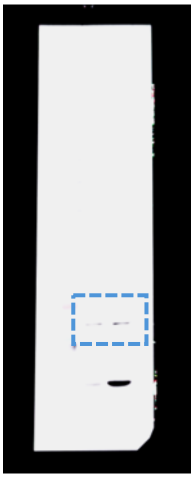

Anti-β-actin

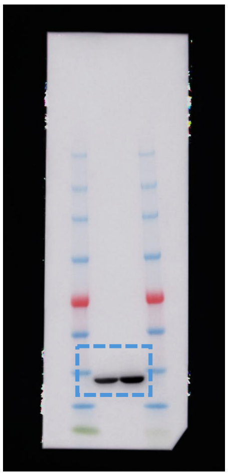

Figure 6c

Anti-SEMA5A

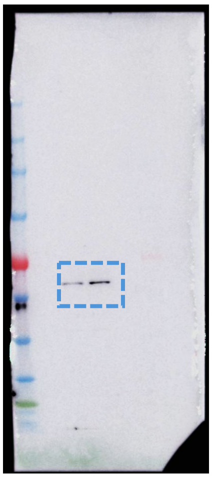

Anti-β-actin

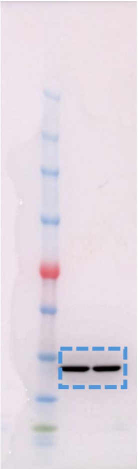

Supplement: Supplementary file 1 — Supplementary Material 1 [file 41598_2025_2840_MOESM1_ESM.pdf]
